# Low Body Weight in Females Is a Risk Factor for Increased Tenofovir Exposure and Drug-Related Adverse Events

**DOI:** 10.1371/journal.pone.0080242

**Published:** 2013-12-02

**Authors:** Cristina Gervasoni, Paola Meraviglia, Simona Landonio, Sara Baldelli, Serena Fucile, Laura Castagnoli, Emilio Clementi, Agostino Riva, Massimo Galli, Giuliano Rizzardini, Dario Cattaneo

**Affiliations:** 1 Department of Infectious Disease, L. Sacco University Hospital, Milan, Italy; 2 Unit of Clinical Pharmacology, L. Sacco University Hospital, Milan, Italy; 3 Clinical Pharmacology Unit, Consiglio Nazionale delle Ricerche Institute of Neuroscience, Department of Biomedical and Clinical Sciences, L. Sacco University Hospital, Università degli Studi di Milano, Milan, Italy; 4 E. Medea Scientific Institute, Bosisio Parini, Italy; Hospital Italiano de Buenos Aires, Argentina

## Abstract

Treatment with tenofovir sometimes leads to non-reversible kidney and/or bone diseases. Factors associated with these drug-related adverse events are poorly characterized. Our objective was to investigate such factors in patients treated long term with daily tenofovir. One-hundred Caucasian HIV-positive patients with basal creatinine clearance >80 mL/min treated with tenofovir for at least 6 months and with at least one assessment of tenofovir plasma trough concentrations were considered. Tenofovir-associated adverse events were defined as the appearance of pathological proteinuria, worsening of renal function or bone demineralization. By multivariate regression analysis, we found that serum creatinine (p = 0.003) and body weight (p = 0.002) were the factors independently associated with plasma tenofovir concentrations. In particular, women with body weight<50 kg had significantly higher plasma tenofovir concentrations than those weighting >50 Kg (160±93 vs.71±52 ng/mL, p<0.001). High tenofovir plasma trough concentrations and the age of the patients were independently associated with the development of drug-related kidney and bone toxicity. In this retrospective study we have shown that HIV-infected women with low body weight are at risk to be exposed to high tenofovir plasma trough concentrations, ultimately resulting in a significant hazard to develop long-term tenofovir complications.

## Introduction

Tenofovir disoproxil fumarate (TDF) is one of the nucleotide reverse transcriptase inhibitors (NRTI) most widely used as fist-line therapy for HIV-infected patients [1]–[4]. TDF is relatively well tolerated in most patients; however episodes of tubular dysfunction (including the development of Fanconi's syndrome), significant decline in glomerular filtration rate and acute kidney injury have often been reported [5]–[7]. These TDF-related kidney disease events may not be reversible [7]. Emerging clinical observations have also revealed considerable relationship between TDF use and loss of bone density in HIV-infected individual [8]–[10]. Taken together, these findings raise serious concerns on the safe use of TDF in clinical practice. The possibility to monitor early exposure of patients to TDF and eventually implement individualized drug dosages has been, however, only poorly explored [11], [12].

Recent investigations have shown that low body weight is an independent risk factor for TDF-associated renal dysfunction in HIV-infected Japanese patients [13], [14]. The Authors hypothesized that small body weight was associated with reduced plasma TDF clearance and thus high plasma TDF concentrations, which could result in renal tubular dysfunction. This hypothesis has been indirectly supported by a retrospective investigation in Caucasian HIV-infected individuals showing significant association between development of kidney impairment and higher TDF plasma trough concentrations [15]. According to the manufacturer's instruction, the recommended TDF dose is 300 mg to be administered once a day in patients with creatinine clearance >50 mL/min and every 48 h in patients with creatinine clearance ranging from 30 to 49 mL/min. No specific indications are usually given in patients with low body weight. These observations suggest that some patients treated with conventional dose regimens may be exposed to high plasma TDF concentrations with an increased risk to develop drug-related adverse events. The identification of factors predisposing to overexposure to TDF may therefore contribute to improve the safety of this drug.

In the present study we: I) evaluated the distribution of TDF plasma trough concentrations in HIV-infected patients treated with TDF at 300 mg qd; II) identified factors associated with TDF plasma concentrations; III) assessed whether high TDF plasma concentrations are independently associated with the development of drug-related toxicity.

## Materials and Methods

### Study population

Male and female HIV-infected patients referred to the Department of Infectious Diseases of the L. Sacco University Hospital with basal creatinine clearance >80 mL/min before starting treatment with TDF were enrolled in the present study. Pediatric subjects, patients with severe hepatic impairment or with history of kidney or bone diseases were excluded from the present study. All patients started TDF treatment at 300 mg given orally every 24 hours (q.d.).

Adherence of patients to therapy was verified through direct questioning during every outpatient visits. Data on self-reported adherence were matched with data from our Pharmacy Department in order to verify that patients effectively picked up the adequate amount of antiretrovirals to fully cover the time between visits. Only patients with high adherence to antiretroviral medications (above 95% of the doses) were considered.

The present study is based on a retrospective analysis of routine TDF pharmacokinetic evaluations carried out as day-by-day clinical practice for the optimization of drug dosing in HIV-infected patients. Written informed consent to patient's management (that is consent for diagnostic evaluations, drug administration and all other medical procedures) was collected prior to his admission to the Department of Infectious Disease of the L. Sacco Hospital. Patients agreed for the use of their records for anonymized future analysis. In compliance with privacy laws, the patients' identification code was encrypted before performing the statistical analyses. Given the retrospective observational nature of the present investigation, no formal approval from the local ethics committee was required according to the legislation of the national drug agency (Agenzia Italiana del Farmaco, available at www.agenziafarmaco.gov.it).

### Study design

In the present retrospective study we considered HIV-infected patients treated with TDF for at least six months and with at least one assessment of TDF plasma trough concentration performed by our laboratory between January 2011 and December 2012 (the measurement of TDF was not available in our hospital before January 2011). Patients already on TDF-based therapy before January 2011 were included in the analysis provided that they fulfilled the inclusion/exclusion criteria.

If more than one TDF concentration was available for each patient we considered only the first drug assessment in the multivariate analysis correlating TDF exposure with drug-related toxicity. Any relevant clinical information on the clinical status of the patient was also recorded (monitoring all adverse events, monitoring of hematological, biochemical and immune-virologic parameters, measurement of vital signs and physical examinations).

TDF related adverse events were defined as episodes of pathological proteinuria, worsening of renal function (at least 30% reduction in the creatinine clearance versus baseline) or bone toxicity (reduced bone mineral density assessed by DEXA in absence of menopause). Creatinine clearance was estimated with the formula of Cockroft-Gault, as follows: GFR =  (140-age)×body weight/serum creatinine×72. In female patients, the formula was multiplied by a constant of 0.85.

### Pharmacokinetic evaluations

Blood samples drawn into EDTA-containing vacutainers® were collected from all patients 24 hours after the last drug intake (a time window of ±5 min was directly verified by the nurse staff and considered as acceptable), immediately before the next TDF administration and placed immediately on ice after drawing. All samples were centrifuged at 3000 g (+4°C), and then plasma was separated and stored at −20°C.

After purification of plasma samples through solid-phase extraction, TDF concentrations were determined using a liquid chromatography method coupled with a mass spectrometry [16] developed and validated according to FDA guidelines [17]. Chromatographic separation was achieved with a gradient (acetonitrile and water with formic acid 0.05%) on a reversed phase analytical column (Atlantis 4.6 mm ×150 mm, Waters, Milan Italy). Detection of TDF was achieved by electrospray ionization mass spectrometry in the positive ion mode.

### Statistical analyses

Results were given as the mean (± standard deviation) or median (plus interquartile range) according to distribution of the data based on results of the Kolmogorov-Smirnov normality test. Multivariate regression analyses were performed using TDF plasma concentrations as the dependent variable, with clinical characteristics as the independent one. Comparisons of demographic, hematological and biochemical parameters, as well as immune-virologic status (CD4 cell count, HIV viral load and co-infection with HCV or HBV) between patients experiencing or not TDF-related side effects were carried out using the unpaired *t*-test or the Mann-Whitney test according to the distribution of the data. The independent association between plasma TDF concentrations and drug-related toxicity was assessed by means of multivariate logistic regression analysis using the event (yes or no) as dichotomic dependent variable (MEDCALC, Software). A p value of less than 0.05 was considered as statistically significant.

## Results

### Patients' characteristics

One-hundred HIV-infected adult patients were included in the present study. Forty-six of them were co-infected with hepatitis C and/or hepatitis B. Patients were at a mean (± standard deviation) of 1042 (±925) days of therapy with TDF/emtricitabine. As shown in Table 1, 63% of them were given concomitantly a boosted protease inhibitor (31 patients were on atazanavir, 14 on lopinavir, 10 on darunavir and 8 fosamprenavir), while the remaining were on efavirenz (15%), nevirapine (4%), or raltegravir (18%).

**Table 1:** Main demographic, haematological and biochemical parameters of HIV-positive patients given tenofovir as part of their maintenance antiretroviral therapy (data were given as mean ± standard deviation).

Parametes	Patients (n = 100)	Women (n = 40)	Men (n = 60)
Tenofovir therapy, days	1042±925	1415±986	824±770
Age, years	45±9	44±8	45±11
Protease inhibitors, %	63	65	69
Non nucleoside reverse transcriptase inhibitors, %	19	20	18
Raltegravir,%	18	15	13
Weight, Kg	62.6±14.0	53.3±12.2	68.6±11.7
Body mass index, Kg/m^2^	21.7±3.8	20.5±4.4	22.5±3.3
Creatinine, mg/dL	0.9±0.7	0.7±0.2	1.0±0.5
Creatinine clearance, mL/min	96.4±36.6	91.1±37.7	100.0±35.9
AST, IU/L	55±76	46±61	61±85
ALT, IU/L	66±133	49±77	78±160
CD4, cells/mL	483±271	488±293	474±254
HIV-RNA, cp/mL	3673±32873	513±1833	5642±41866
Pts with HIV-RNA >100 cp/mL,%	16	17	15
HCV coinfection, %	31	30	33
HBV coinfection, %	12	13	9
HCV+HBV coinfection, %	3	3	4

### Distribution of TDF plasma trough concentrations

**Figure 1:**
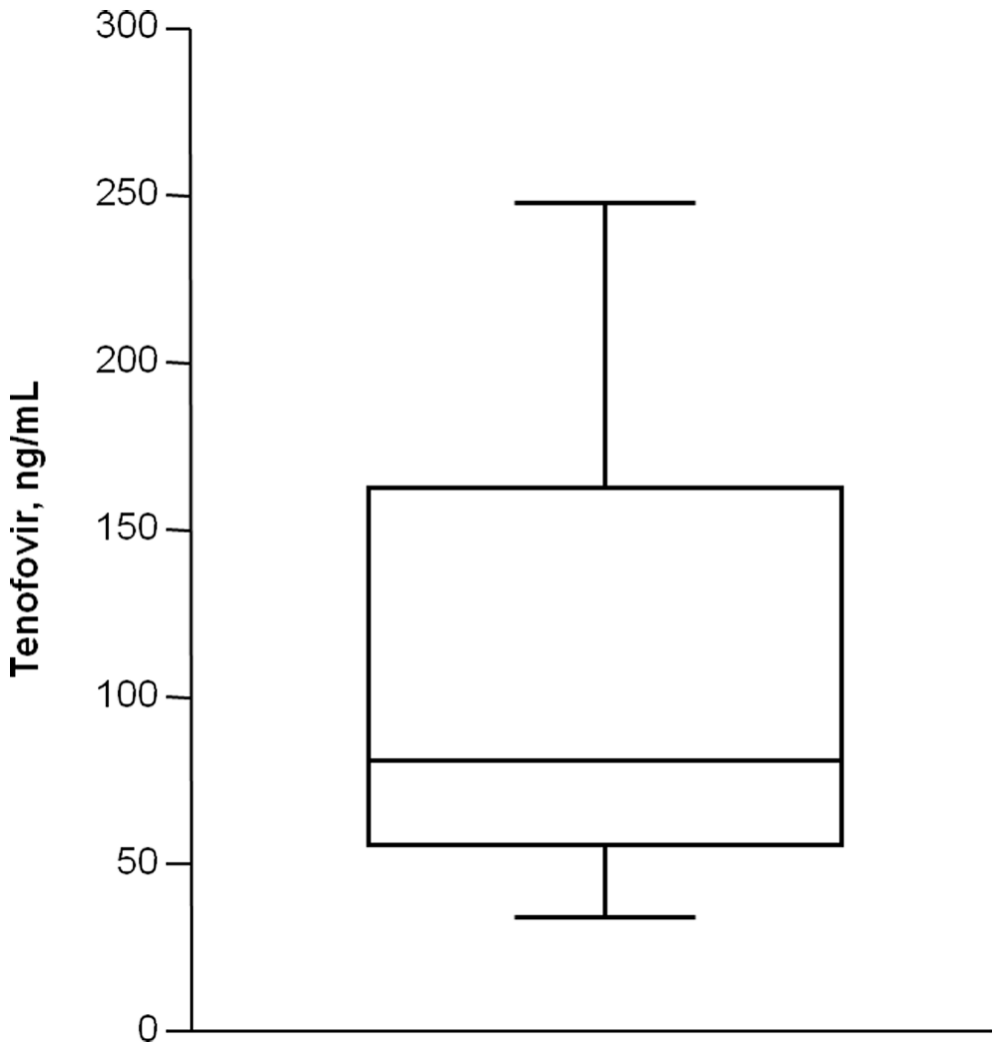
Box-plot of Tenofovir (TDF) plasma trough concentrations measured in 100 HIV-infected patients.

Overall, a wide distribution in the measured TDF plasma trough concentrations was observed, with values ranging from 20 to 452 ng/mL (Figure 1). This distribution was associated with an inter-patient variability in the plasma TDF concentrations of 89.1%. Intrapatient variability, estimated in patients with at least 3 assessments of TDF concentrations (n = 37), was 36.0%.

### Clinical factors associated with TDF plasma concentrations

**Figure 2:**
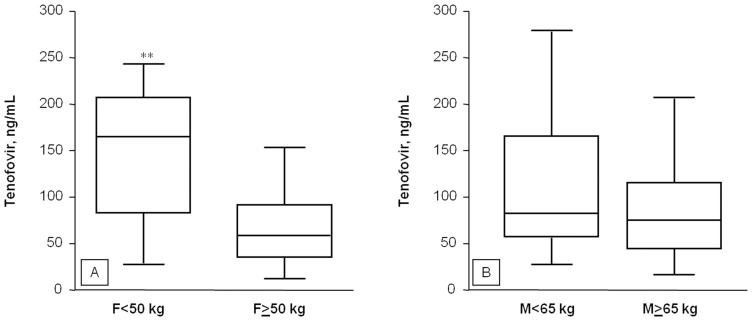
TDF plasma trough concentrations measured female (panel A) and male (panel B) HIV infected patients stratified according to median body weight (**p<0.01).

By multivariate regression analysis (Table 2), the factors independently associated with plasma TDF concentrations were serum creatinine (p = 0.003) and body weight (p = 0.002). This trend was confirmed also when using creatinine clearance (p = 0.002) and body mass index (p = 0.005) instead of serum creatinine and body weight, respectively. In order to further investigate the relationship between body weight and TDF plasma concentrations we repeated the statistical analysis stratifying the patients by gender and by weight using the median values as cutoff (50 kg for women and 65 kg for men). Using this approach we found that women with body weight<50 kg (n = 20) had significantly higher plasma TDF concentrations than those weighting ≥50 Kg (n = 20, 160±93 vs.71±52 ng/mL, p<0.001, Figure 2, panel A). This association was not observed in men (Figure 2, panel B).

**Table 2:** Multivariate regression analysis of variables independently associated with tenofovir plasma trough concentrations.

Variable	Correlation coefficient	p-value
Gender	0.1085	0.976
Age	0.2350	0.708
Concomitant ARV drugs	−0.1244	0.253
Body weight	−0.2635	0.002
Serum creatinine	0.2480	0.003
AST, IU/L	0.0351	0.900
ALT, IU/L	0.0190	0.751
Hepatitis C co-infection	−0.1004	0.250

ARV: antiretroviral.

No difference was found in the TDF plasma trough concentrations when stratifying data according to concomitant antiretroviral drugs (120.5±88.6 vs. 94.3±55.6 vs. 106.6±71.2 ng/mL in patients concomitantly treated with PI, NNRTI and RAL, respectively). Similarly, no age-related differences in plasma TDF concentrations were found in our HIV-infected adult patients (data not shown).

### TDF plasma concentrations and drug-related toxicity

Twenty-six out of the 100 HIV-positive patients enrolled in the present study experienced either kidney (n = 11), or bone disease (n = 11) or both complications (n = 4). The comparison of demographic, hematochemical and clinical characteristics of HIV-infected patients that did (n = 26) or did not (n = 74) develop TDF-related adverse events is shown in Table 3. Patients experiencing TDF-related toxicity had significantly higher plasma drug concentrations (145±103 vs. 101±67 ng/mL, p = 0.016), were significantly older than those with no TDF-related toxicity (48±11 vs. 43±10 years, p = 0.031) and showed a non-significant trend for longer exposure to TDF. The independent association between TDF plasma trough concentrations and drug-related toxicity was confirmed also by multivariate logistic regression analysis (p = 0.008). No significant associations between immuno-virologic status or HCV/HBV co-infection and TDF-related toxicity were found.

**Table 3:** Comparison of demographic, haematological and biochemical parameters of HIV-positive patients that did (n = 26) or did not (n = 74) develop tenofovir-related adverse events (data were given as mean ± standard deviation).

Parameters	Toxicity YES (n = 26)	Toxicity NO (n = 74)	P-value
Tenofovir therapy, days	1340±927	945±909	0.068
Tenofovir conc., ng/mL	145±103	101±67	0.016
Male gender, %	61.5	57.9	0.781
Age, years	48±11	43±10	0.031
Protease inhibitors, %	69	61	0.235*
Non nucleoside reverse transcriptase inhibitors, %	19	19	
Raltegravir,%	12	20	
Weight, Kg	63±15	62±14	0.815
Body mass index, Kg/m^2^	22.5±4.8	21.4±3.4	0.206
AST, IU/L	48±45	58±85	0.555
ALT, IU/L	53±77	71±149	0.573
CD4, cells/mL	553±292	453±257	0.102
HIV-RNA, cp/mL	13140±64067	301±1334	0.087
Pts with HIV-RNA >100 cp/mL, %	12	18	0.265*
Hepatitis C co-infection, %	46	45	0.907

* chi square test.

Overall, 40 HIV-infected women with median body weight of 50 kg (ranging from 36 to 83 kg) and median age of 46 years (ranging from 26 to 68 years) were enrolled in the present study. Thirty-two percent of women with body weight ≤50 kg vs. 18% of women with body weight >50 kg experienced TDF-related toxicity. Women experiencing toxicity had TDF plasma concentrations significantly higher than those who did not experience drug related adverse events (173±131 vs. 106±65 ng/mL, p = 0.042), with no differences concerning patients 'age (43±7 vs. 44±8 years, p = 0.656). Such associations were not found in men (data not shown).

## Discussion and Conclusion

This is the first retrospective, observational study showing that women, but not men, with low body weight have the highest risk of being overexposed to TDF plasma concentrations, ultimately increasing their risk of developing renal and/or bone complications. The following indirect clinical evidence supports this observation. A cross-sectional survey in a French hospital-based cohort involving 2500 HIV-infected patients showed a high prevalence of renal impairment that was significantly associated with female gender, age, body mass index and use of TDF [18]. The predictive role of low body mass index and female gender on the development of renal dysfunction was also confirmed in cohorts of HIV-infected patients from Tanzania [19] and from Europe [20].

As additional findings, we documented that patients treated with TDF at 300 mg/daily showed a wide distribution of TDF plasma trough concentrations, and confirmed that renal function and body weight were the two major sources for the observed interindividual variability of TDF pharmacokinetics, as previously reported [21]–[23]. These observations underline the importance of close monitoring for renal and bone function in patients with low body weight treated with conventional TDF dose regimens [13], [14].

More recently, a retrospective analysis from the Swiss HIV Cohort study has shown that TDF with either boosted protease inhibitors (lopinavir or atazanavir) lead to a greater initial decline in estimated GFR than TDF with efavirenz [24]. This result has been related to a drug-to-drug interaction with protease inhibitors resulting in a 30% increase in plasma levels of TDF and a consequent greater increase in the renal damage [25]. In our study we found no significant differences in the TDF plasma trough concentrations between patients taking protease inhibitors, efavirenz/nevirapine or raltegravir. The lack of significant association between TDF plasma levels and concomitant HAART medications was also confirmed by multivariate regression analysis. Similarly, concomitant use of protease inhibitors vs. NNRTI was not independently associated with the development of TDF-related adverse events. The discrepancy between our and previous results can be only apparent and due, at least in part, to the different periods of observation in the two studies. In the study by Young and co-workers [24] the differences between protease inhibitors and NNRTI on GFR decline, significant in the first 6 months of therapy, did attenuate thereafter; this is in line with our study that involved patients being at more than 6 months of therapy with TDF.

In the cohort we studied, we found that patients experiencing TDF-related adverse events were significantly older, with significantly higher TDF plasma trough concentration, and with longer exposure to TDF than patients that did not experience drug toxicity. Our data are in agreement with previous findings in a large cohort of adults HIV-infected subjects [15], showing that patients with kidney tubular dysfunction had significantly higher TDF plasma trough concentrations than those with normal renal function. Taken together, these results suggest an association between high TDF plasma concentrations and the development of long-term TDF-related complications (irreversible renal insufficiency and/or bone mineral loss). Given the retrospective nature of our investigation, we are not able to conclude whether it is chronic overexposure to TDF that determined reduced glomerular filtration rate or, conversely, that the progressive development of renal insufficiency resulted in an accumulation of TDF that is excreted through glomerular filtration. Moreover, given the limited number of HIV-infected women included in the statistical analyses, we were not able to provide definitive conclusions but, rather, a working hypothesis to be further investigated in prospective, adequately powered clinical trials. We believe, however, that these results also show that therapeutic monitoring of TDF plasma trough concentrations, taken as surrogate marker of systemic drug exposure, may allow the early identification of patients at risk of toxicity related to overexposure to TDF. In these patients modified dosing regimens (i.e. dose-interval adjustments) could be eventually pursued with the goal to reduce adverse events related to high levels of TDF exposure.

In summary, High TDF plasma trough concentrations and patients' age were independently associated with the development of drug-related kidney and bone toxicity. HIV-infected women with low body weight are at risk to be exposed to high TDF plasma trough concentrations, ultimately resulting in a significant hazard to develop drug-related complications. This could be eventually handled by routine therapeutic monitoring of TDF plasma concentrations.

Results of our study also confirmed the emerging evidence that HIV-infected female subjects are more susceptible to adverse events related to antiretroviral treatment [25], [26]. In order to optimize treatment in all HIV-infected patients, there is the compelling need for specific studies and female participation in both cohort studies and clinical trials should be promoted.
